# Psychosocial Aspects of Bruxism: The Most Paramount Factor Influencing Teeth Grinding

**DOI:** 10.1155/2014/469187

**Published:** 2014-07-13

**Authors:** Mieszko Wieckiewicz, Anna Paradowska-Stolarz, Wlodzimierz Wieckiewicz

**Affiliations:** ^1^Division of Dental Materials, Faculty of Dentistry, Wroclaw Medical University, 26 Krakowska Street, 50425 Wroclaw, Poland; ^2^Department of Maxillofacial Orthopedics and Orthodontics, Faculty of Dentistry, Wroclaw Medical University, 26 Krakowska Street, 50425 Wroclaw, Poland; ^3^Department of Prosthetic Dentistry, Faculty of Dentistry, Wroclaw Medical University, 26 Krakowska Street, 50425 Wroclaw, Poland

## Abstract

In clinical practice, patients suffering from an occlusal parafunctional activity have increased. It can be observed that a negative influence of environment aggravates patient's health. The aim of this paper is to present the impact of environment and development of human civilization on the prevalence of bruxism and the correlation between them. The authors grasp the most relevant aspects of psychological and anthropological factors changing over time as well as their interactions and describe a relationship between chronic stress and bruxism. Current literature shows how contemporary lifestyle, working environment, diet, and habits influence the patient's psychoemotional situation and the way these factors affect the occluso-muscle condition.

## 1. Introduction

Bruxism is an oral habit consisting of involuntary rhythmic or spasmodic nonfunctional gnashing, grinding, or clenching of teeth, unlike chewing movements of the mandible, which may lead to occlusal trauma [1]. The term “la bruxomanie” was used for the first time by Maria Pietkiewicz in 1907 [2].

Research shows that the most dangerous form of this pathology is night bruxism, which has a psychoemotional and occlusal origin [3, 4]. If not treated, it leads to damage of the teeth, periodontium and oral mucosa, pathology of the muscles constituting the masticatory system, headache and cervical pain, temporomandibular, and hearing disorders [5]. Stress related disturbances, including depression and anxiety, are a real problem in a highly developed society. Clinical studies suggest that stress is the main reason for patients to seek medical advice (50–75%) [6–8]. This is also confirmed by the large number of medicines used in order to treat stress related problems in western countries such as antidepressants, anxiolytics, and hypnotics, which decrease arterial blood pressure and the level of cholesterol [9]. Moreover, the number of patients seeking treatment because of temporomandibular disorders and oral parafunctions is increasing, which may confirm a correlation between these conditions and a growing number of chronic stressors in highly developed societies. This type of disorder can also be observed in younger people and it is more common in females [10–13]. It is generally accepted that chronic stressful situations and mental diseases conduct to the development of occlusal parafunctions and temporomandibular disorders without being the only cause. Additional reasons include interceptive occlusal contacts, malocclusions, traumas/microtraumas, hormone disorders, rheumatism, orthopedic problems, and masticatory system inflammations [14–16]. A rapid development of this disease at the turn of the 20th and 21st centuries induced the authors to attempt to find a cause of this process.

The aim of this paper is to show the correlation between chronic stress, development of civilization, and bruxism based on current literature.

## 2. Materials and Methods

Literature is available from PubMed, PubMed Central, and CINAHL databases that were published between 1955 and 2014. Valuable original and review articles related to the terms bruxism, stress, occlusal parafunctions, temporomandibular disorders, psychology, anthropology, and sociology have been selected for this paper.

## 3. Results

### 3.1. Psychological Aspects of Bruxism

According to Selye [24], stress involves a biological strain of an organism, which is caused by various somatic and/or mental stimuli. These stimuli are called stressors. “Being under stress” means that a person is under an influence of unspecified stimuli, which are revealed by specific changes characterizing this situation. Stressors, regardless of their type, stimulate in an organism stereotypical, nonspecific, and complex adaptation reactions. This adaptation is controlled by hormonal and neurohormonal processes. A state of strain of an organism is called stress, and it can be divided into acute and chronic. Chronic stress belongs to the most destructive factors threatening a human organism [25].

Servan-Schreiber et al. [26–29] present the pathophysiology of stress in a different way. They report a so-called “emotional brain,” which has a completely separate structure from the neocortex and it functions independently. This “brain” is located in the limbic system, so in the central part of the encephalon, and it consists of three main anatomical elements: the hippocampal gyrus, cingulate gyrus, and amygdala. These elements have a far less complicated structure than the neocortex; that is, they are not arranged in regular bundles of neurons, but the nerve cells are rather mixed here. The “emotional brain” controls vital emotions and reactions. Pathological chronic stress and emotional disorders result from functional disturbances of the “emotional brain,” which most often are a consequence of traumas and/or family and professional life. All impressions concerning external environment are processed in the brain. The central nervous system is responsible for their assessment. Signals reach the limbic system and hypothalamus, where they trigger proper emotions and stimulate the sympathetic nervous system releasing adrenaline, which i.a. leads to faster breathing and heartbeat, a higher muscle tension, and an increased sugar level and blood pressure. Any external information which triggers such a response may be recognized as a stressor [30, 31]. Our ancestors usually manifested this analysed biological warning reaction in a form of motor activity. After surviving the danger, their bodies functioned normally again. A contemporary individual, living in a highly developed society, has been deprived of these reaction possibilities. The effects of suppressing emotions and motor activities burden the function of an organism resulting in several neuromuscular disorders [32].

Various pathological emotional experiences more and more often result in the development of a muscular parafunction/bruxism. This can be related to occlusion or can be caused entirely by psychological stimulation. It had been proved that compulsive, controlling, and aggressive persons are more vulnerable to develop bruxism [33]. This disorder involves unconscious teeth clenching and grinding, which leads to gradual damage of the dentition and periodontium, damage of the oral mucosa, increased tension and hypertrophy of masticatory muscles, chronic headaches and cervical pain, and abnormality of the temporomandibular joints as well as hearing problems [18–22]. The most important symptoms related to bruxism are presented in Table 1. Suggestions that teeth grinding is connected with malocclusions do not seem to be true. Dental patients (5–30%) suffer from this type of disorders occurring in the temporomandibular joint, whereas 50–75% of society needs to be treated orthodontically, at least to a moderate degree [23]. Accordingly, this malocclusion is classified as peripheral factor influencing bruxism [2]. Bruxism is a parafunction in which the contraction of the temporal and masseter muscles is responsible for clenching of the dental arches, while the contraction of the pterygoid muscles is responsible for lateral movements, potentially affecting the temporomandibular joints [34]. The most destructive type of this disorder is sleep bruxism [35]. This condition is associated with a rhythmic activity of the masticatory muscles, characterized by a repeated contraction of the masticatory muscles. The highest activity is observed during the phase of sleep called REM (rapid eye movement sleep). In this phase, several parts of the encephalon, including the limbic system, are very active [36]. According to literature, average activity of muscles is several times more intensive in patients with bruxism than in patients showing no bruxism symptoms [36–38]. The intensity of contractions during sleep considerably exceeds a maximum patients' ability to clench their teeth when they are aware of it [39]. Based on the etiology, bruxism can be divided into awake, sleep, occlusion-dependent, psycho-dependent, and a mixed type depending both on the occlusion and psyche. Based on the mandible movements, bruxism is classified as centric, lateral eccentric, anterior eccentric, mixed eccentric, and extra eccentric [40].

**Figure 1:**
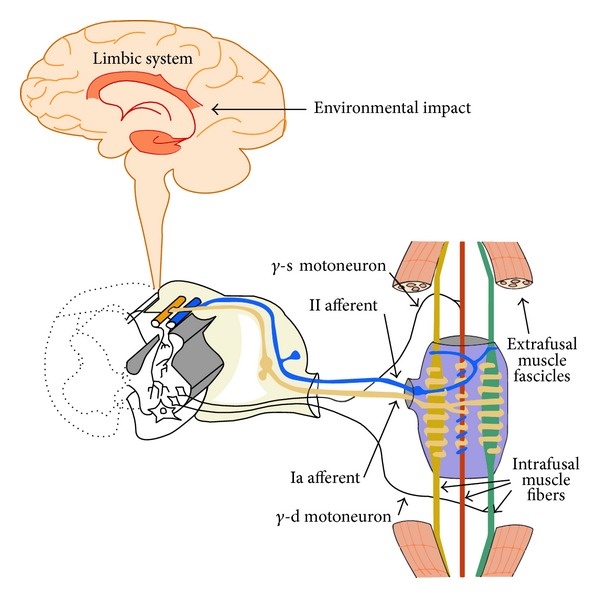
Schematic diagram of the gamma loop, which is permanently stimulated by external impulses in a group of people affected by chronic stress [17].

A muscle, when passive, stays under constant tension resulting from the alpha neurons stimulation by the central stream of impulses. This tension does not lead to muscle fatigue. The main role in the tension coordination belongs to gamma neurons, which are controlled by higher centers and participate in the development of an abnormal muscle activity (Figure 1) [17]. Chronic stress and warning reactions triggered by it manifest themselves as functional deficiencies of the nervous-muscle system and are the main etiologic factors of psycho-dependent bruxism [41]. When these disturbances appear, attention is drawn by venting the accumulated tensions directly through the dental arches, by means of increased muscle activity, which may lead to headaches. This abnormal activity and increased tension are controlled by the limbic system and hypothalamus, stimulated by chronic stress, which are connected with the cortex. In this way, through the reticular formation of the brainstem occurs a correlation between stimulating awareness, vegetative activities, and affective emotional behavior along with the muscle tension. Central stimulations increase a segment reflex activity mainly over stimulation of gamma motor neurons. The increased reflex activity causes muscle tone, which may vary from a state of higher tension up to rigidity. The gamma loop is activated through the descending reticulospinal tract, which results in increased muscle tone. The possibility of an influence of the higher centers can lead to an abnormal increase of muscle tone, which explains the occurrence of higher muscle activity triggered by psychological stimuli [42–44].

### 3.2. Social Aspects of Bruxism

According to Young [45], the rapid development of civilization is an apparent factor conducive to diseases, which do not pose a direct threat to human life. The author describes the changes in the dentition of native inhabitants of Australia, which occurred after contact with western culture. Aborigines' oral hygiene improved considerably, but changes of eating habits and consumption of a large amount of food with high sugar content as well as soft diet caused an unexpected increase of teeth crowding and dental caries. A change in lifestyle and diet prevented the dentition from severe attrition. Occlusal-muscular disorders were quite rare. Because of changes in nutritional habits, for example, softer diet at the expense of raw meat and vegetables, nowadays contemporary inhabitants of Australia use different and less muscle groups than the primitive tribes or the inhabitants in the 19th century. For this reason, contemporary inhabitants suffer from similar functional disorders of the stomatognathic system, regardless of their background. Watson [46] studied the changes in dentition of the inhabitants of north-west Mexico and showed the impact of diet and agricultural factors on the health of teeth tissue. The dentition of this group clearly shows that the development of civilization reduced the physiological wear-and-tear of the teeth, which followed the changes in nutrition. Moreover, the author suggests that psychological factors and a diet of the contemporary people inhabiting this region are causative factors of changes of a different type, that is, caries and noncaries defects.

The studies explicitly revealed that a new form of losing teeth must be connected with a different impact of contemporary environment on the stomatognathic system. Omnipresent chronic stressors, to which a human organism is subjected every day, lead indirectly to various disorders. The study of Bayar et al. [47] proves that occlusal parafunctions are closely connected with psychological disturbances of different degrees of severity, most of which are caused by an inability to accept everyday reality or by exaggeration of experiencing external stimuli. Bayar assumes that the etiopathogenesis of bruxism is complex; nevertheless, he indicates that the most important is the psychological factor, which is also confirmed by other authors [48, 49]. The studies of Bracha et al. [50] and Gungormus and Erciyas [51] distinguish from 3 many emotional disorders those which are accompanied by occluso-muscle disorders, that is, excessively experienced stress, depression, neurosis, phobias, personality disorders, anxiety, and paranoid states. These diseases are common in highly developed societies, in which surrounding environment directly leads to their occurrence. Chronic stress, lack of sleep, rest time, and activities are conducive to the development of psychoemotional disorders, vascular diseases, dermatological problems, gastric disturbances, and neuromuscular disorders [52–55]. Omnipresent chronic stressors in a contemporary society are called civilization stress [56]. Independent of mental disorders, availability and overuse of stupefying agents may result in an increase of parafunctional muscular activity. This problem was proved by Gomez et al. [57], whose study confirms a correlation between drug addiction and an increase of abnormal occluso-muscle function.

Manfredini et al. [58] show that significant factors in the development of occlusal parafunctions are malocclusions and abnormal bites. It should be stressed that an occlusal aspect most often connected with psychological disorders gives the picture of full-blown bruxism. In everyday clinical practice, patients who are diagnosed with occlusal problems often do not show symptoms characteristic of emotional disturbances. Only after taking the patient's medical history are revealed problems often related to the family and/or professional life. It should be emphasized that a properly conducted medical interview as well as a physical examination is an important part of therapy of the temporomandibular disorders.

A change of profile of health threats as well as a longer lifespan in contemporary societies caused dissemination of diseases and treatment methods, which were very rare or even did not exist in the past. An example of such change is the orthodontic treatment, whose rapid development took place in the second part of the 20th century. Chu et al. [59] confirm this thesis in their study, which is based on a group of 240 students, 41% of whom undergo/underwent orthodontic treatment. Nonetheless, occluso-muscle parafunctions belong to a group of disorders, which affect an increasing part of a society. Frequently, these parafunctions are a cause of dental treatment failure. Kinsel and Lin [60] studied the prosthetic complications in 152 patients with implant borne fixed dental prostheses and proved seven times higher odds in bruxers.

Other studies reveal that a frequency of occluso-muscle disorders also depends on ethnic background. Hicks et al. [61] conducted studies concerning the frequency of bruxism among American students of different backgrounds. The results showed higher bruxism prevalence in students of Asian origin, an average among European and Latin-American group and the lowest in the Afro-American group. Several authors proved that psycho-dependent occlusal parafunctions more and more often concern children under 10 years [62–64]. These studies reveal that occluso-muscle disorders affect people of different ages. However, bruxism is more common in females [2].

## 4. Discussion

The etiology of bruxism is multifactorial. Occlusion abnormalities, chronic stress, and mental disorders are responsible for this condition. It should be highlighted that there is a tendency of more frequent occurrence of oral parafunctions in highly developed societies. Thus, it seems that the occlusal aspect in the development of bruxism is of minor importance. This is confirmed by studies in which bruxism is observed in patients with implant borne restorations, among them there is no impulsation from the periodontium [65]. Symptoms of bruxism may be unnoticeable for a longer period. Only after having experienced a nagging pain does a patient start to seek medical help. Patients often consult doctors of different specialties, especially neurologists or ophthalmologists, but also ENT specialists, since one of the symptoms of severe bruxism can be a sleep apnea, resulting in a long-term state of chronic fatigue and increasing stress related to “not keeping up” with projects. Bruxism functions as a kind of* perpetual motion machine*, as intensifying symptoms resulting from the abnormal functioning of an organism increase a feeling of being stressed, and in consequence leads to an increased muscle tone and teeth grinding [66, 67]. In its stage, this problem is often ignored by patients, which has serious consequences in the form of destruction of the dentition and severe abnormalities of muscles and joints. In this phase, the condition requires at least two-phased prosthetic treatment. It also calls for multidisciplinary treatment, in which specialists like a dentist, psychiatrist, neurologist, psychologist, physiotherapist, and a dental technician should participate. Worldwide discussions and studies of scientists resulted in recognizing sleep bruxism as a disorder, which was then included in the International Classification of Sleep Disorders in 2005 [67].

## 5. Conclusions

On the basis of the data collected by the authors based on current literature it can be stated that the prevalence of bruxism depends on the development of civilization and the modern lifestyle. In this way the psychological aspect of occluso-muscle disorders becomes more significant. Contemporary environment is full of incessant stress threats and thus dangerous to our health and life. In recent years, the number of patients suffering from bruxism has increased significantly. For this reason doctors should pay more attention to this parafunction in order to diagnose it at an early stage.

## Figures and Tables

**Table 1:** Possible symptoms of bruxism according to medical disciplines [18–23].

Branch of medicine	Symptoms observed
Dentistry	Clenching or grinding of the teeth while asleep (often noticed by sleeping partner); hypersensitivity of teeth to hot, cold, sweet, and so forth; attrition; fractures of teeth; negative consequences in periodontium/gingival recessions; loss of teeth; damages and cracks of fixed and removable dentures (especially dental ceramics); cheek and tongue biting

Otolaryngology	Ear sounds (tinnitus), ear aches (referred pain) with possible hearing loss, ear infections, apnea

Neurology	Constant, dull headache; pain in the temples; sleep disorders (insomnia); anxiety, stress, and depression; dizziness; vertigo

Ophthalmology	Hypersensitivity to light, pain in the eye or around the eye, difficulties in sight focus

Physical Therapy	Sore jaw muscles, facial pain or jaw pain, higher muscle tension, myofascial pain, temporomandibular joint disorders (clicking), trismus, hand and arm tingling

Others	Changes in facial appearance, eating disorders
